# COVID-19 booster doses reduce sex disparities in antibody responses among nursing home residents

**DOI:** 10.1007/s40520-025-02990-0

**Published:** 2025-03-08

**Authors:** Oladayo A. Oyebanji, Anna Yin, Nicholas Sundheimer, Vaishnavi Ragavapuram, Patrick Shea, Yi Cao, Philip A. Chan, Aman Nanda, Rohit Tyagi, Sakeena Raza, Nadia Mujahid, Yasin Abul, Alejandro B. Balazs, Jürgen Bosch, Christopher L. King, Sabra L. Klein, Stefan Gravenstein, David H. Canaday, Brigid M. Wilson

**Affiliations:** 1 Division of Infectious Diseases and HIV Medicine, Case Western Reserve University School of Medicine, Cleveland, OH USA; 2 Johns Hopkins University Bloomberg School of Public Health, Baltimore, MD USA; 3 Center for Global Health and Diseases, Case Western Reserve University, Cleveland, OH USA; 4 Ragon Institute of MGH, MIT, and Harvard, Cambridge, MA USA; 5 Division of Infectious Diseases, Warren Alpert Medical School of Brown University, Providence, RI USA; 6 Division of Geriatrics and Palliative Medicine, Warren Alpert Medical School of Brown University, Providence, RI USA; 7 Center of Innovation in Long-Term Services and Supports, Veterans Administration Medical Center, Providence, RI USA; 8 Brown University School of Public Health Center for Gerontology and Healthcare Research, Providence, RI USA; 9 Geriatric Research Education and Clinical Center (GRECC), VA Northeast Ohio Healthcare System, Cleveland, OH USA

**Keywords:** Antibody response, Sex differences, Aging, Omicron, Nursing home residents, Older adults, Immunosenescence

## Abstract

**Background:**

Data suggest that antibody responses following COVID-19 vaccines are a correlate of protection. Some studies, including the clinical trials of COVID-19 mRNA vaccines, did not stratify and evaluate whether antibody responses to COVID-19 vaccines differed between the sexes or with aging. This gap in research is particularly relevant for older populations such as nursing home residents (NHR). We hypothesized that sex differences in vaccine-induced antibody responses may intersect with age and be diminished among older adults residing in nursing homes.

**Methods:**

We analyzed serum samples from 638 NHRs collected serially after the primary two-dose series and three subsequent booster doses of mRNA SARS-CoV-2 vaccinations. We analyzed anti-Spike IgG and neutralizing antibody titers to the Wuhan and Omicron BA.4/5 variant strains. Mixed-effects models predicting log-transformed titers were estimated to compare responses across vaccine doses, focusing on sex-differential responses. For detected post-dose sex differences, additional sample times were analyzed to assess the duration of the difference.

**Results:**

Following the primary series, female NHRs with a prior history of SARS-CoV-2 infection had significantly higher Wuhan anti-Spike antibodies and neutralizing antibody titers than male NHRs with differences persisting up to nine months post-vaccination. Subsequent monovalent booster doses and a bivalent booster dose eliminated this disparity. We did not detect any differential response to the Omicron BA.4/5 variant.

**Conclusions:**

The blunting of sex differences in antibody response observed following the primary series by the 1st booster dose underscores the importance of booster vaccination in this population.

**Supplementary Information:**

The online version contains supplementary material available at 10.1007/s40520-025-02990-0.

## Background

Coronavirus disease 2019 (COVID-19) vaccines have played a vital role in mitigating the global impact of the pandemic. Examining the intricacies of vaccine efficacy and safety, an increasingly important factor has come to light - the role of biological sex differences in the immunological response to these vaccines, particularly among vulnerable populations such as nursing home residents (NHRs). Historically, there has been an acknowledgment of the substantial influence of sex differences on immune responses to various infections and vaccines [1–4]. The emergence of the COVID-19 pandemic and the subsequent rollout of vaccines have provided a unique opportunity to explore these disparities in greater detail, often overlooked in clinical trials [5–7].

Previous studies suggest that sex-based differences in the immune response to COVID-19 may significantly affect disease outcomes [8–10]. However, we lack comprehensive studies focusing on this topic within the specific context of NHRs. Generally, females exhibit heightened inflammatory, antiviral, and humoral immune responses compared to males, with roles for genes and sex steroid hormones, like estradiol [11, 12]. Similarly, the immune response in older persons, especially females, shows a progressive decline, highlighting the intricate interaction of sex and age in immune function [13–15]. This decline in immune function with aging, known as immunosenescence, particularly affects the efficacy of vaccines and increases susceptibility to infections and poorer health outcomes in older populations. Furthermore, sex differences in COVID-19 outcomes, with males exhibiting more severe illness, have prompted investigation into differential immune responses [16–18]. These findings collectively emphasize the need for a nuanced understanding of sex-specific immunological responses to COVID-19 vaccines among distinct populations such as institutionalized older adults.

In a previous study involving a cohort of NHRs and healthcare workers, we showed that female NHRs elicited a higher T-cell response than male NHRs following repeated mRNA vaccinations [19]. This current study investigates whether there are variations in humoral immune responses to COVID-19 vaccines between male and female NHRs. We aim to contribute to the growing body of knowledge surrounding biological sex differences in humoral responses to COVID-19 vaccines, specifically focusing on NHRs, who are susceptible to severe COVID-19 outcomes due to their advanced age and underlying health conditions.

## Methods

### Ethical approval

This study was approved by the Western-Copernicus Group Institutional Review Board with protocol number STUDY20211074. All participating residents or their legally authorized representatives provided informed consent to be enrolled. The study is in accordance with the 1964 Declaration of Helsinki and its later amendments.

### Study design and study population

**Fig. 1:**
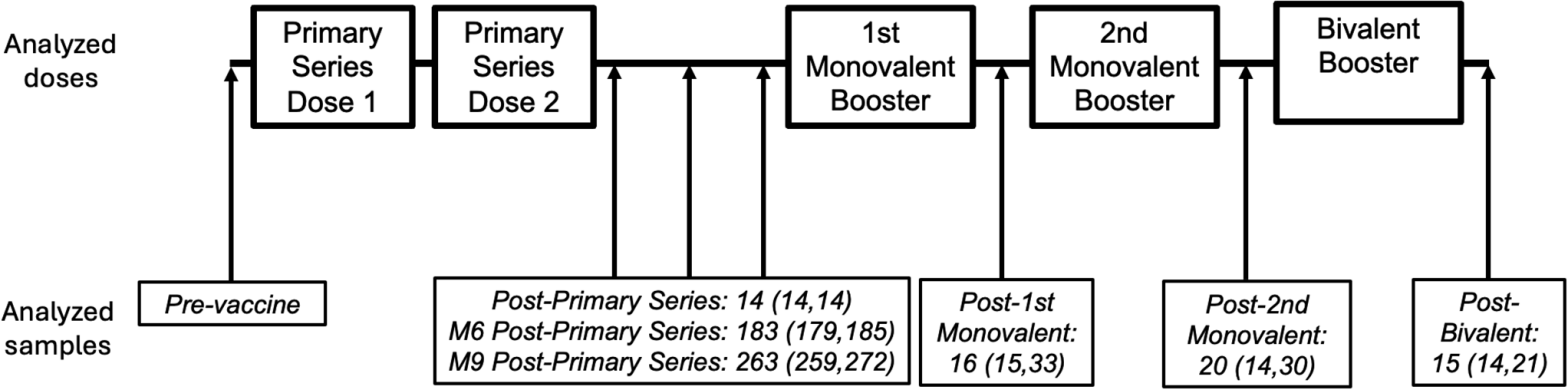
Timeline of analyzed doses and days to sampling. Serum samples were collected serially from participants after mRNA vaccination. This analysis focuses on the post-vaccine draws following a 2-dose mRNA Primary Series and three subsequent booster doses. The monovalent boosters are Wuhan-based while the bivalent booster contains Wuhan and Omicron BA4/5. Post-vaccine draws and the median (IQR) days following vaccine dose are indicated. Not all analyzed subjects received all listed doses. M6: Month 6, M9: Month 9

The current analysis is part of an ongoing study [20–24] in which NHRs are consented and serially sampled before and after each SARS-CoV-2 vaccine dose. Participants were recruited from 18 nursing homes in Ohio and 16 nursing homes in Rhode Island. Comorbidity and functional status were added to the study data collection, based on chart review of subject health records at the time of enrollment, after the study began. Thus, these variables were collected for most, but not all, of the subjects. Residents who received SARS-CoV-2 mRNA vaccines [(BNT162b2 (Pfizer-BioNTech) or mRNA-1273 (Moderna)] were included, and those who received other vaccines were excluded. Participants typically received their first monovalent booster dose 8–9 months after the primary vaccination series, and their second monovalent booster 4 to 6 months after the first booster. In this current study, we report results from blood samples obtained at time points following vaccination: approximately 14 days, 6 months, and 9 months post-primary vaccination series; and 14 days post-first, second monovalent booster, and post-bivalent booster (Fig. 1). The primary vaccination series and the first and second monovalent boosters were Wuhan-based mRNA vaccines while the bivalent booster consisted of Wuhan and Omicron BA.4/5 strains. All samples were collected between December 2020 and December 2022. In the setting of breakthrough infection during the study, the subject’s samples collected from the breakthrough through the next vaccine dose were excluded from this analysis.

Participants were deemed “infection prior” if they had a prior SARS-CoV-2 infection at the time of each sampling based on: (1) Prior documentation in their medical chart of a positive polymerase chain reaction (PCR) or antigen test; or (2) An increase in SARS-CoV-2 antibody levels beyond variation of the assay, that could not be explained by vaccination e.g. rise in spike-specific and N-antigen-specific antibodies.

### Anti-spike assay

We assessed vaccine-induced antibody response using bead-multiplex immunoassay for anti-spike for SARS-CoV-2 wild-type (Wuhan-Hu-1, “Wuhan”) strain and BA.4/5 variants as previously described [20]. Stabilized full-length spike protein (aa 16-1230, with furin site mutated and recombinant SARS-CoV-2 S(1-1208)-2P-3 C-His8-TwinStrep) from Wuhan and SARS-CoV-2 S-2P(1-1208)-3 C-His8-TwinStrep BA.5 from Omicron BA.4/5 variants and full-length N (aa1-419) from Wuhan, obtained from the Frederick National Laboratory (FNL) were conjugated to magnetic microbeads (Luminex) and Magpix assay system (BioRad, Inc). Anti-Wuhan spike IgG levels were measured and calculated in Binding Antibody Units (BAU)/mL based on the FNL standard, and anti-spike BA.4/5 are shown in arbitrary units (AU)/mL. Values between 0 and 1 were considered to be 1, reflecting the assumed precision of the assay.

### SARS-CoV-2 pseudovirus neutralization assay

We produced lentiviral particles pseudotyped with spike protein based on the Wuhan and BA.4/5 strains as previously described to define the neutralizing activity of vaccine recipients’ sera against coronaviruses [25]. We performed three-fold serial dilutions that ranged from 1:12 to 1:8748 and added 50–250 infectious units of pseudovirus for 1 h. 50% pseudoviral neutralizing antibody titers (pNT50) values were calculated by taking the inverse of the 50% inhibitory concentration value for all samples with a pseudovirus neutralization value of 80% or higher at the highest serum concentration. The lower limit of detection (LLD) of this assay is 1:12 dilution.

### Statistical analysis

Our cohort was summarized overall and by vaccine dose, illustrating the changing size and makeup of the vaccinated and sampled subjects (Table 1, Supplemental Table 1). Age distributions and time since prior vaccination dose were summarized as medians with interquartile ranges (IQR) (Table 1; Fig. 1). Categorical variables were summarized with counts and percentages. Differences in functional status and comorbidities between male and female subjects were summarized using standardized mean differences to quantify imbalance between the sex groups.

**Table 1:** Demographics of nursing home residents (NHRs), overall and by vaccine dose

		Any analyzed dose	Post-primary series	Post-1st monovalent booster	Post-2nd monovalent booster	Post-bivalent booster
Female	N subjects	308	60	157	120	133
	Age	78 (70,88)	83 (73, 88.5)	78 (70, 86)	78.5 (69.8, 88)	76 (68, 87)
	Race/Ethnicity: White Non-Hispanic	254 (82%)	52 (87%)	127 (81%)	97 (81%)	106 (80%)
	Race/Ethnicity: Black Non-Hispanic	49 (16%)	6 (10%)	28 (18%)	20 (17%)	26 (20%)
	Race/Ethnicity: Hispanic	3 (1%)	1 (2%)	1 (1%)	2 (2%)	0 (0%)
	Race/Ethnicity: Other/Missing	2 (1%)	1 (2%)	1 (1%)	1 (1%)	1 (1%)
	Prior infection	-	28 (47%)	81 (52%)	83 (69%)	101 (76%)
Male	N subjects	330	92	224	81	128
	Age	74 (67, 83)	75 (70, 83)	74 (68, 83)	74 (66, 81)	73 (66, 78.25)
	Race/Ethnicity: White Non-Hispanic	258 (78%)	79 (86%)	177 (79%)	60 (74%)	92 (72%)
	Race/Ethnicity: Black Non-Hispanic	61 (18%)	13 (14%)	39 (17%)	17 (21%)	31 (24%)
	Race/Ethnicity: Hispanic	5 (2%)	0 (0%)	4 (2%)	2 (2%)	2 (2%)
	Race/Ethnicity: Other/Missing	6 (2%)	0 (0%)	4 (2%)	2 (2%)	3 (2%)
	Prior infection	-	41 (45%)	113 (50%)	49 (60%)	92 (72%)

Separate models predicting vaccine response by dose, sex, prior infection, and all interactions of these 3 variables were estimated for each combination of strain (Wuhan and BA.4/5) and assay (anti-spike antibodies and neutralizing titers). As some subjects were sampled repeatedly, mixed-effects linear regression models predicting log-transformed titers were estimated to adjust for correlated outcomes within subjects using random intercepts. Model assumptions were checked and marginal mean sex differences were tested using model contrasts for each dose combination and prior SARS-CoV-2 infection.

For doses with detected sex differences post-vaccination, we analyzed additional samples from the post-vaccination subjects obtained before the next dose to test if the observed sex differences persisted over time using the modeling approach described above. Samples obtained 150–210 days and 240–300 days post-vaccine were grouped as 6-month and 9-month post-dose samples, respectively.

For those subjects with infections before the post-primary series sample, we compared the available dates of prior infections between males and females using a Wilcoxon rank sum test. To assess for possible sex differences in attrition over time due to death, we identified subjects with a study withdrawal due to death in the year following primary series vaccination and compared death rates by sex using a Fisher’s exact test. To assess for possible sex differences before Primary Series vaccination, we compared the geometric mean titers (GMT) of males and females with available samples using a t-test of the log-transformed titers, stratifying tests on assay, and prior infection.

A sensitivity analysis was performed, adding further adjustments to the models predicting post-dose log-transformed titers across four vaccine doses. In these models, we considered age as a covariate and facility as an additional random effect with subjects nested within nursing home facilities. The results of these models addressing possible confounding factors were compared to the previously described models.

Results were considered statistically significant at a two-sided alpha of 0.05. All analyses were performed in R version 4.2.2 using smd, nlme and emmeans packages for summaries, models and contrast estimation.

## Results

Our study involved 638 NHRs in total throughout the study and reports on 60 or more NHRs of each sex for each of the 4 vaccine doses. The study cohort changed over time due to new enrollment and discharge from NH or withdrawal/death, and vaccines received and sample availability varied among subjects. Subjects were followed longitudinally to the extent that their enrollment, vaccination, and availability for sample draws permitted. Across all time points, the participants were predominantly of white ethnicity. Female NHRs had a median age of 76 to 83 and were older than the male NHRs, with a median age range of 73 to 75 (Table 1). In addition to advanced age, this cohort had a high burden of comorbidities and reduced functional status (Supplemental Table 1). Comorbidities and functional status were not collected from some of the earliest enrolled subjects in our study; thus, these data are missing for 29% of the post-primary series cohort. Among subjects with available functional status, many more male NHRs were completely independent. Among those in the cohort with available comorbidity data, we observed higher rates of chronic obstructive pulmonary disease (COPD) and heart failure among males. Female NHRs had more dementia while the rates of diabetes mellitus and immunosuppressive illnesses or immunomodulatory medications were similar between sexes (Supplemental Table 1). After the primary vaccination series, similar rates of SARS-CoV-2 infection were observed across both sexes at each vaccine dose (Table 1).

We detected sex differences in antibody responses following the primary vaccination series among subjects with prior SARS-CoV-2 infection such that the observed geometric mean titer (GMT) of Wuhan anti-Spike antibodies in females was 3.2 times higher than that of males (2431 vs. 755, model-adjusted ratio = 2.83, *p* = 0.007) and the GMT of Wuhan neutralizing titers was 2.8 times that of males (1742 vs. 623, model-adjusted ratio = 2.61, *p* = 0.004) (Table 2). These sex differences were not detected before initial vaccination among subjects with available pre-vaccine samples (*n* = 151), though pre-vaccine responses were often at or below the assay lower limits of detection, and higher GMTs were observed in female than male subjects with prior infection (Supplemental Fig. 1). The same subset of dose and prior infection status were found to have significant sex differences when considered in sensitivity models attempting to adjust for potential confounders with available data (Supplemental Table 2).

**Fig. 2:**
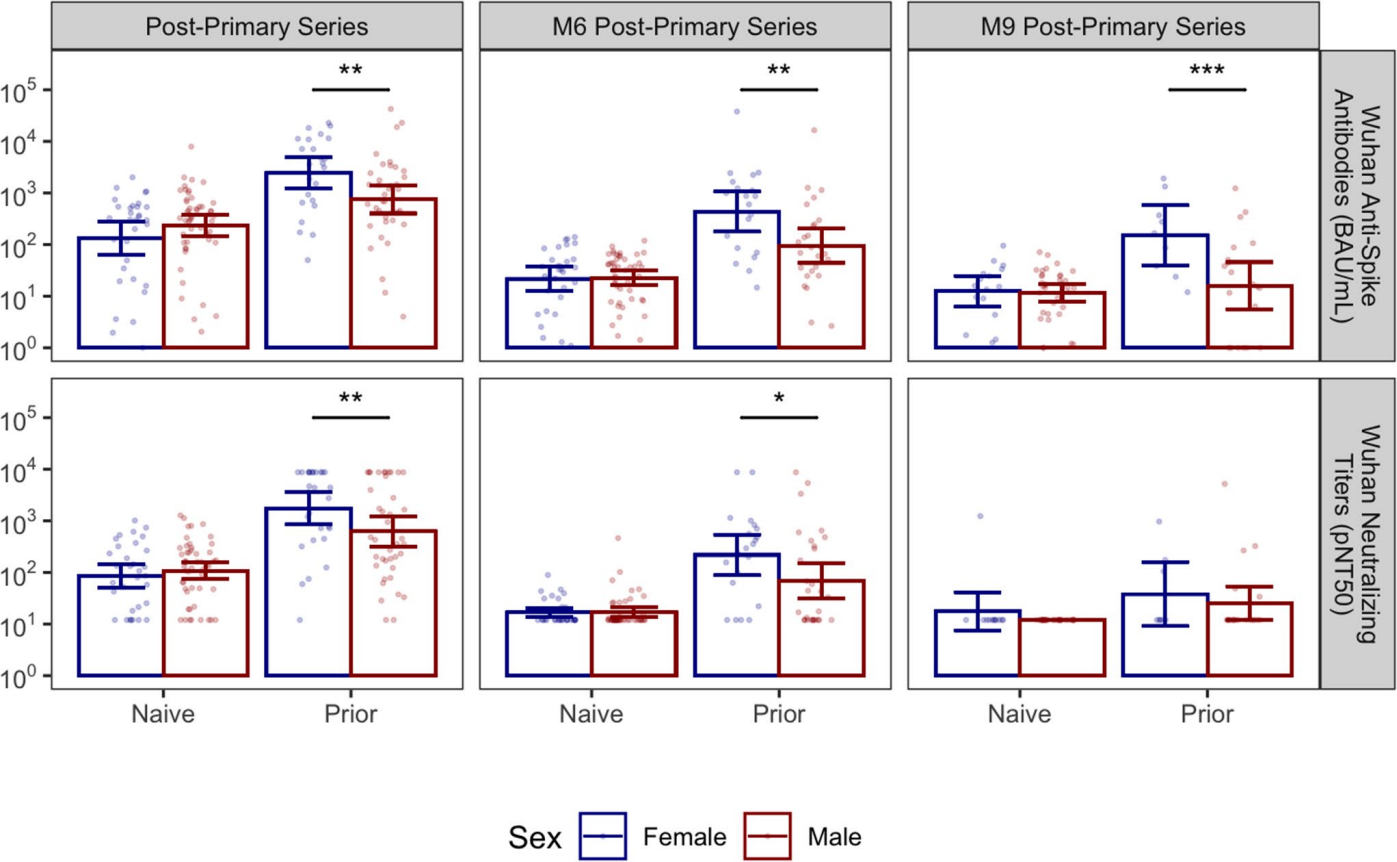
Anti-Wuhan Spike and neutralizing antibody titers over time among female and male NHRs. The bar graphs show the kinetics of anti-Spike (upper panel) and neutralizing (lower panel) antibodies against the Wuhan strain across different time points among female and male NHR. Wuhan anti-Spike is measured in BAU/mL. The lower limit of detection of the neutralization assay was 1:12, while the upper limit was 1:8748. Post-primary series sera were taken 2–4 weeks after the 2nd vaccine dose, completing the primary series, while M6 and M9 post-primary series sera were taken 6–8 months and 7–10 months later, respectively. Bars and whiskers show GMT with 95% CI. Blue: Female, Red: Male. Naive subjects: no prior infection, Prior subjects (previously infected). Male and female subjects were compared with model contrasts after estimating mixed-effects linear models predicting log-transformed titer with the interaction of sex, prior infection, and sample time within strain and assay. *, **, *** are significance levels of model contrasts with *p* < 0.05, < 0.01, < 0.001 respectively. BAU/ml: Binding Antibody Unit/ml, pNT50: Pseudoneutralization titer 50, NHR: Nursing Home Residents, GMT: Geometric Mean Titer, CI: Confidence Interval

Post-vaccination differences persisted and were statistically significant when examined in a linear mixed-effects model, adjusted for repeated samples within subjects across vaccine doses. When focusing on the post-vaccination time points before the first monovalent booster in residents with post-primary series data (*n* = 152) using similar models, we found that the sex-based difference in immunological response among prior-infected subjects persisted at 6 months (model estimated geometric mean titer ratio (GMTR) = 4.2, *p* = 0.003) and 9 months for the spike protein (model estimated GMTR = 16.3, *p* < 0.001) and at 6 months for neutralization titers (model estimated GMTR = 2.8, *p* = 0.014) (Fig. 2). Among prior-infected NHRs sampled post-primary series, there was no statistically significant difference in the time elapsed since prior SARS-CoV-2 infection between males and females with medians of 87 days and 84 days, respectively (Wilcoxon *p* = 0.55). Among 4 mortality events in this post-primary series cohort observed in the year following primary series vaccination, 3 were men.

**Fig. 3:**
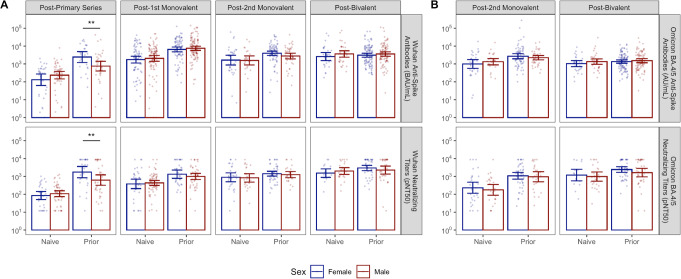
Anti-Spike and Neutralizing Antibody titers against Wuhan (Panel A) and Omicron BA.4/5 (Panel B) strains across booster doses among female and male NHR. The bar graph shows the post-vaccination anti-Spike and neutralizing antibody titers against the Wuhan and Omicron strains across the boosters among female and male NHR. Wuhan anti-Spike is measured in BAU/mL. Note, all figures show post-boost titers except those for the post-primary series. The lower limit of detection of the neutralization assay was 1:12, while the upper limit was 1:8748. Post-vaccination sera were taken 2–4 weeks after each dose. Bars and whiskers show GMT with 95% confidence intervals. Blue: Female, Red: Male. Naive subjects: no prior infection, Prior subjects: previously infected. Male and female subjects were compared with model contrasts after estimating mixed-effects linear models predicting log-transformed titer with the interaction of sex, prior infection, and vaccine dose within strain and assay. *, **, *** are significance levels of model contrasts with *p* < 0.05, < 0.01, < 0.001 respectively. BAU/ml: Binding Antibody Unit/ml per milliliter for Wuhan and arbitrary units (AU)/mL for BA.4/5, pNT50: Pseudoneutralization titer 50, NHR: Nursing Home Residents, GMT: Geometric Mean Titer, CI: Confidence Interval

We did not detect sex differences in vaccine response as measured by these assays to any of the three booster doses examined stratified by prior SARS-CoV-2 infection; nor did we detect sex differences in infection-naive subjects following the primary vaccination series (Fig. 3A). We also did not detect sex differences when comparing Omicron BA.4/5 anti-Spike antibodies and neutralizing titers for the second monovalent booster and first bivalent booster doses (Fig. 3B).

**Table 2:** GMT by assay, vaccine dose, prior infection status for male & female nursing home residents with model-estimated ratio female to male and model p-values

Vaccine dose	Strain	SARS-CoV-2 Status	Assay	GMT (95% CI), Female	GMT (95% CI), Male	Crude Ratio, F/M	Adjusted Ratio (95% CI), F/M	ModelP-value
Post-Primary Series	Wuhan	Naive	Neutralizing Titer	85 (50,143)	108 (74,158)	0.79	0.96 (0.53,1.75)	0.892
			Anti-Spike Antibody	131 (62,275)	234 (145,378)	0.56	0.67 (0.35,1.29)	0.227
		Prior	Neutralizing Titer	1742 (844,3595)	623 (319,1218)	**2.8**	**2.61** **(1.35**,**5.02)**	**0.004**
			Anti-Spike Antibody	2431 (1212,4875)	755 (403,1413)	**3.22**	**2.83** **(1.34**,**5.99)**	**0.007**
Post-1st Monovalent	Wuhan	Naive	Neutralizing Titer	381 (207,701)	433 (314,596)	0.88	0.97 (0.56, 1.65)	0.897
			Anti-Spike Antibody	1745 (1115,2731)	2055 (1414,2986)	0.85	0.87 (0.55,1.37)	0.551
		Prior	Neutralizing Titer	1336 (786,2271)	995 (666,1487)	1.34	1.36 (0.77,2.41)	0.291
			Anti-Spike Antibody	6521 (4772,8912)	7521 (5786,9774)	0.87	0.83 (0.54,1.29)	0.408
Post-2nd Monovalent	Wuhan	Naive	Neutralizing Titer	891 (503,1579)	827 (484,1413)	1.08	0.9 (0.45,1.79)	0.76
			Anti-Spike Antibody	1642 (870,3098)	1571 (877,2815)	1.05	0.93 (0.44,1.97)	0.851
		Prior	Neutralizing Titer Titer	1427 (1043,1952)	1271 (856,1886)	1.12	1.25 (0.66,2.35)	0.489
			Anti-Spike Antibody	3979 (3018,5246)	2769 (1914,4006)	1.44	1.3 (0.77,2.2)	0.326
	Omicron BA.4/5	Naive	Neutralizing Titer	232 (113,475)	177 (89,353)	1.31	1.14 (0.51,2.54)	0.742
			Anti-Spike Antibody	990 (562,1743)	1333 (888,2000)	0.74	0.68 (0.38, 1.2)	0.181
		Prior	Neutralizing Titer	1074 (697,1654)	957 (498,1840)	1.12	1.34 (0.66,2.71)	0.41
			Anti-Spike Antibody	2707 (1958,3744)	2247 (1705,2961)	1.2	1.05 (0.69,1.58)	0.828
Post-Bivalent	Wuhan	Naive	Neutralizing Titer	1532 (884,2656)	2015 (1305,3111)	0.76	0.89 (0.4,1.97)	0.774
			Anti-Spike Antibody	2605 (1556,4360)	3630 (2404,5480)	0.72	0.72 (0.35,1.49)	0.38
		Prior	Neutralizing Titer	2975 (2118,4178)	2277 (1371,3782)	1.31	1.02 (0.58,1.81)	0.937
			Anti-Spike Antibody	3100 (2414,3980)	3626 (2736,4807)	0.85	0.92 (0.6,1.41)	0.714
	Omicron BA.4/5	Naive	Neutralizing Titer	1187 (558,2524)	982 (539,1789)	1.21	1.63 (0.66,3.98)	0.279
			Anti-Spike Antibody	1046 (707,1547)	1354 (956,1917)	0.77	0.71 (0.41,1.24)	0.224
		Prior	Neutralizing Titer	2434 (1709,3466)	1630 (936,2840)	1.49	1.44 (0.74,2.79)	0.271
			Anti-Spike Antibody	1361 (1104,1678)	1522 (1165,1989)	0.89	0.92 (0.66,1.28)	0.622

Anti-Spike Antibodies are measured in Binding Antibody Unit (BAU)/ml for Wuhan strain and Arbitrary Units (AU)/mL for BA.4/5; Neutralizing Titer is measured in 50% Pseudovirus Neutralizing Antibody Titers (pNT50). GMT: Geometric Mean Titer, CI: Confidence Interval, F: Female, M: Male

## Discussion

Understanding biological sex differences in the immune response to vaccination may help optimize vaccine efficacy and develop targeted interventions. Our study investigated the potential influence of biological sex on the humoral response to COVID-19 mRNA vaccines among NHRs.

Notably, we observed a significant sex-based disparity in antibody levels following the primary vaccination series among prior SARS-CoV-2-infected residents, with females exhibiting substantially higher levels of Wuhan anti-spike antibodies and neutralizing titers compared to their male counterparts. While this aligns with some evidence highlighting sex-based differences in immune responses to viral infections and vaccinations [26–28], it remains unclear why this disparity was not present among the SARS-CoV-2 infection-naive residents in our study, as reported by Shapiro et al. [29]. This may have been influenced by factors such as different ages between naïve and prior residents, and comorbidities, among others. However, similar to our findings, in a large multicenter study of NHRs, Trevisan et al. did not observe any sex differences in antibody response among naive residents who received 2 doses and prior-infected residents who received only 1 dose of the mRNA vaccines [30]. In contrast to the vaccination regimen in that study, the prior-infected residents in our study received 2 doses of the mRNA vaccine. This additional antigenic exposure could have provided a window to amplify the disparity between the sexes in our study.

This disparate increase in antibody response among prior-infected female residents persisted up to 9 months after the primary vaccination series, as observed in studies that reported sex differences in antibody response among this population [29]. This observation highlights the sustained impact of sex on vaccine-induced antibody production and suggests that the sex-based differences are not merely transient but may persist over an extended period after primary vaccination having implications on vaccine effectiveness and durability [31]. Remarkably, sex-based differences in the persistence of antibody responses to influenza vaccination were associated with variations in the longevity of vaccine-induced immunity between males and females [32–34]. Thus, this persistence of differences in the immune response to COVID-19 vaccination may have implications for the duration of protection against SARS-CoV-2, especially in high-risk populations such as NHRs.

Interestingly, these sex-based differences in antibody response were not observed following booster doses, regardless of prior SARS-CoV-2 infection. This absence of sex differences in response to booster doses suggests that additional doses may effectively bridge potential immune response gaps between sexes and equalize immunity in both sexes [6, 29, 35]. This leveling effect of the booster doses has also been reported among younger populations, where disparities in vaccine-induced antibody response due to age and sex, after the initial primary vaccination series, were found to be mitigated by a booster dose [36, 37]. Similarly, we did not detect differences between sexes to the Omicron BA.4/5 variant for the second monovalent booster and first bivalent booster doses.

Our study found similar rates of prior SARS-CoV-2 infection and time elapsed since COVID-19 infection in both sexes across each vaccine dose. While we see a trend for a higher pre-vaccination GMT among previously infected women than in men, this difference did not reach statistical significance (Anti-Spike p-value = 0.17; Neutralizing Titer p-value = 0.063, Supplemental Fig. 1). This offers a reasonable explanation for the prior infection difference because the women started at a higher antibody titer. Shapiro, et al. previously reported that before vaccination with influenza, older women start the respiratory viral season at higher influenza antibody levels than older men [38]. It is well established that females including older populations generally mount more robust and durable immune responses to infections and vaccinations that can result in these higher titers than men [39, 40].

We note important limitations of our study. Sex-specific comorbidities and frailty can influence antibody response among institutionalized older adults [29, 30]. We did not collect comorbidities and functional status for some of the early enrollees in our study. Among those with available comorbidity data, most post-primary series male subjects we recruited lived in a state Veterans home. This presents both a distinct population and a different electronic health record for review than males in other community nursing homes. We still need to interpret findings at this timepoint with caution, as comorbidities may be confounders that impact vaccine response [41]. Also, we did not explore potential biological mechanisms that underlie the observed sex differences, such as the role of sex hormones and genetic factors, nor could we account for lifetime differences in prior exposure to respiratory viruses. Females differ in traditional caregiver roles in raising children and grandchildren and have been reported to present more often with clinical viral respiratory infections than males [42] and therefore may have different lifetime immune priming, including, perhaps other beta coronaviruses. Such a scenario could explain both differences in severity with SARS-CoV-2 infections [43] as well as differential initial boosting with the primary series, i.e., different anamnestic responses. Thus we speculate that historical viral exposure could provide a pool of memory cells available for a booster response to primary SARS-CoV-2 vaccination or infection. Also, Sex differences observed in our NHR population with prior infection could reflect a sex difference in the survivorship bias driven by higher mortality in male NHRs than in NHR females infected in the pre-vaccine era [44]. Future research should delve deeper into these aspects to better understand the reasons behind these differences.

In conclusion, our study underscores the dynamic nature of sex-based differences in vaccine response and emphasizes the significance of booster doses in reducing these disparities. While a sex disparity was initially observed after the primary series, booster vaccinations effectively mitigated differences. These findings have implications for optimizing vaccine strategies in vulnerable populations and provide insight into the influence of prior infection on vaccine response in this specific context. Further research is required to unravel these sex-based differences in underlying mechanisms and implications and investigate their impact on COVID-19 outcomes.

## Electronic supplementary material

Below is the link to the electronic supplementary material.

Supplementary Material 1

## Data Availability

Persons can contact the authors for access to the dataset.
