# Supplementary material for: COVID-19 booster doses reduce sex disparities in antibody responses among nursing home residents
Source: Aging Clin Exp Res. 2025 Mar 8;37(1):73. doi: 10.1007/s40520-025-02990-0 (PMC11889018; doi:10.1007/s40520-025-02990-0)
Supplement: Supplementary file 1 — Supplementary Material 1 [file 40520_2025_2990_MOESM1_ESM.docx]

**Supplemental Table 1: Functional status and medical history, by dose and sex**

| **Functional Status** | **All doses** | | | **Post-Primary Series** | | | **Post-1st Monovalent Booster** | | | **Post-2nd Monovalent Booster** | | | **Post-Bivalent Booster** | | |
| --- | --- | --- | --- | --- | --- | --- | --- | --- | --- | --- | --- | --- | --- | --- | --- |
|  | **Female** (N=308) | **Male** (N=330) | **SMD** | **Female** (N=60) | **Male** (N=92) | **SMD** | **Female** (N=157) | **Male** (N=224) | **SMD** | **Female** (N=120) | **Male** (N=81) | **SMD** | **Female** (N=133) | **Male** (N=128) | **SMD** |
| Complete independence | 32  (16.2%) | 36  (18.0%) | 0.19 | 5  (17.2%) | 19 (37.3%) | 0.66 | 10  (10.2%) | 26 (18.3%) | 0.35 | 17  (18.7%) | 6 (10.9%) | 0.51 | 19  (19.6%) | 21 (24.1%) | 0.39 |
| Modified Independence | 45  (22.7%) | 55  (27.5%) |  | 14 (48.3%) | 17 (33.3%) |  | 30  (30.6%) | 48 (33.8%) |  | 14  (15.4%) | 16 (29.1%) |  | 19  (19.6%) | 26 (29.9%) |  |
| Supervision | 11  (5.6%) | 9  (4.5%) |  | 0  (0.0%) | 2  (3.9%) |  | 4  (4.1%) | 3  (2.1%) |  | 3  (3.3%) | 4  (7.3%) |  | 7  (7.2%) | 3  (3.4%) |  |
| Minimal assistance | 18  (9.1%) | 17  (8.5%) |  | 3  (10.3%) | 4  (7.8%) |  | 13  (13.3%) | 14  (9.9%) |  | 9  (9.9%) | 2  (3.6%) |  | 7  (7.2%) | 6  (6.9%) |  |
| Moderate assistance | 38  (19.2%) | 31  (15.5%) |  | 4  (13.8%) | 3  (5.9%) |  | 25  (25.5%) | 24 (16.9%) |  | 16  (17.6%) | 7  (12.7%) |  | 18  (18.6%) | 8  (9.2%) |  |
| Maximal assistance | 34  (17.2%) | 38  (19.0%) |  | 3  (10.3%) | 5  (9.8%) |  | 10  (10.2%) | 20 (14.1%) |  | 19  (20.9%) | 14 (25.5%) |  | 18  (18.6%) | 16 (18.4%) |  |
| Total assistance | 20  (10.1%) | 14  (7.0%) |  | 0  (0.0%) | 1  (2.0%) |  | 6  (6.1%) | 7  (4.9%) |  | 13  (14.3%) | 6  (10.9%) |  | 9  (9.3%) | 7  (8.0%) |  |
| *Not obtained* | 110 | 130 |  | 31 | 41 |  | 59 | 82 |  | 29 | 26 |  | 36 | 41 |  |
| **Comorbidities/ History** |  | | | | | | | | | | | | | |  |
| Cardiovascular diseases | 89  (31.3%) | 137 (44.2%) | 0.27 | 3  (8.3%) | 43 (59.7%) | 1.29 | 39  (24.8%) | 98 (43.8%) | 0.41 | 41  (34.2%) | 28 (34.6%) | 0.01 | 46  (34.6%) | 55 (43.0%) | 0.17 |
| Dementia | 53 (18.7%) | 34 (11.0%) | 0.22 | 13 (36.1%) | 12 (16.7%) | 0.45 | 23 (14.6%) | 28 (12.5%) | 0.06 | 29 (24.2%) | 6 (7.4%) | 0.47 | 23 (17.3%) | 12  (9.4%) | 0.23 |
| Pulmonary diseases | 78  (27.5%) | 93  (30.0%) | 0.06 | 3  (8.3%) | 31 (43.1%) | 0.87 | 36  (22.9%) | 67 (29.9%) | 0.16 | 33  (27.5%) | 18 (22.2%) | 0.12 | 39  (29.3%) | 34 (26.6%) | 0.06 |
| Genitourinary diseases | 66  (23.2%) | 72  (23.2%) | 0.00 | 6  (16.7%) | 16 (22.2%) | 0.14 | 31  (19.7%) | 53 (23.7%) | 0.10 | 30  (25.0%) | 18 (22.2%) | 0.07 | 38  (28.6%) | 33 (25.8%) | 0.06 |
| Diabetes Mellitus | 93  (32.7%) | 109 (35.2%) | 0.05 | 14 (38.9%) | 29 (40.3%) | 0.03 | 55  (35.0%) | 74 (33.0%) | 0.04 | 40  (33.3%) | 29 (35.8%) | 0.05 | 44  (33.1%) | 47 (36.7%) | 0.08 |
| Hypercoagulable conditions (DVT or PE, others) | 30  (10.6%) | 21  (6.8%) | 0.13 | 1  (2.8%) | 4  (5.6%) | 0.14 | 18  (11.5%) | 14  (6.3%) | 0.18 | 19  (15.8%) | 6  (7.4%) | 0.27 | 19  (14.3%) | 9  (7.0%) | 0.24 |
| Cancer - actively receiving treatment | 3  (1.1%) | 2  (0.6%) | 0.04 | 0  (0.0%) | 1  (1.4%) | 0.17 | 3  (1.9%) | 2  (0.9%) | 0.09 | 2  (1.7%) | 0  (0.0%) | 0.18 | 2  (1.5%) | 1  (0.8%) | 0.07 |
| Cancer - remote history | 36 (12.7%) | 47 (15.2%) | 0.07 | 3  (8.3%) | 19 (26.4%) | 0.49 | 25 (15.9%) | 33 (14.7%) | 0.03 | 16 (13.3%) | 8 (9.9%) | 0.11 | 16 (12.0%) | 14 (10.9%) | 0.03 |
| Immunosuppressive illness | 17  (6.0%) | 14  (4.5%) | 0.07 | 3  (8.3%) | 3  (4.2%) | 0.17 | 7  (4.5%) | 9  (4.0%) | 0.02 | 9  (7.5%) | 3  (3.7%) | 0.17 | 10  (7.5%) | 5  (3.9%) | 0.16 |
| Immunomodulatory medications | 2  (0.7%) | 2  (0.6%) | 0.01 | 0 | 0 | 0.00 | 1  (0.6%) | 2  (0.9%) | 0.03 | 1  (0.8%) | 0  (0.0%) | 0.13 | 0 | 0 | 0.00 |
| *Not obtained* | 24 | 20 |  | 24 | 20 |  | 0 | 0 |  | 0 | 0 |  | 0 | 0 |  |

Cardiovascular diseases include heart failure, coronary artery disease, atrial fibrillation or flutter, and prosthetic heart valves. Pulmonary diseases include chronic obstructive pulmonary disease, asthma, and pulmonary fibrosis. Genitourinary diseases include chronic kidney disease or recurrent urinary tract infections. Immunosuppressive illnesses include human immunodeficiency virus and autoimmune conditions. Immunomodulatory medications include steroids, monoclonal antibodies, etc. DVT - Deep Venous Thrombosis, PE - Pulmonary Embolism. Percents are based on the number of subjects with data obtained. SMD - Standardized Mean Difference.

**Supplemental Table 2: NH Wuhan sensitivity model results, with model-estimated ratio of female-to-male and model p-values; model includes age as covariate and residents clustered within facility**

| **Vaccine dose** | **SARS-CoV-2**  **Status** | **Assay** | **Adjusted Ratio**  **(95% CI), F/M** | **Model P-value** |
| --- | --- | --- | --- | --- |
| Post-Primary Series | Naive | Neutralizing Titer | 0.6 (0.3, 1.2) | 0.146 |
|  |  | Anti-Spike Antibody | 0.85 (0.44, 1.64) | 0.617 |
|  | Prior | Neutralizing Titer | **2.37 (1.09, 5.11)** | **0.029** |
|  |  | Anti-Spike Antibody | **2.09 (1.05, 4.15)** | **0.036** |
| Post-1st Monovalent | Naive | Neutralizing Titer | 0.78 (0.49, 1.25) | 0.301 |
|  |  | Anti-Spike Antibody | 0.86 (0.48, 1.52) | 0.595 |
|  | Prior | Neutralizing Titer | 0.83 (0.53, 1.3) | 0.415 |
|  |  | Anti-Spike Antibody | 1.1 (0.61, 1.99) | 0.756 |
| Post-2nd Monovalent | Naive | Neutralizing Titer | 0.8 (0.38, 1.69) | 0.563 |
|  |  | Anti-Spike Antibody | 0.79 (0.39, 1.6) | 0.513 |
|  | Prior | Neutralizing Titer | 1.3 (0.77, 2.18) | 0.322 |
|  |  | Anti-Spike Antibody | 1.12 (0.59, 2.12) | 0.724 |
| Post-Bivalent | Naive | Neutralizing Titer | 0.62 (0.3, 1.28) | 0.199 |
|  |  | Anti-Spike Antibody | 0.72 (0.32, 1.63) | 0.43 |
|  | Prior | Neutralizing Titer | 0.91 (0.6, 1.4) | 0.681 |
|  |  | Anti-Spike Antibody | 0.79 (0.44, 1.43) | 0.437 |

Anti-Spike Antibodies are measured in Binding Antibody Unit (BAU)/ml for Wuhan strain and Arbitrary Units (AU)/mL for BA.4/5; Neutralizing Titer is measured in 50% Pseudovirus Neutralizing Antibody Titers (pNT50). GMT: Geometric Mean Titer, CI: Confidence Interval, F: Female, M: Male

**Supplemental Figure 1: Pre-vaccination titers, by infection status and sex**


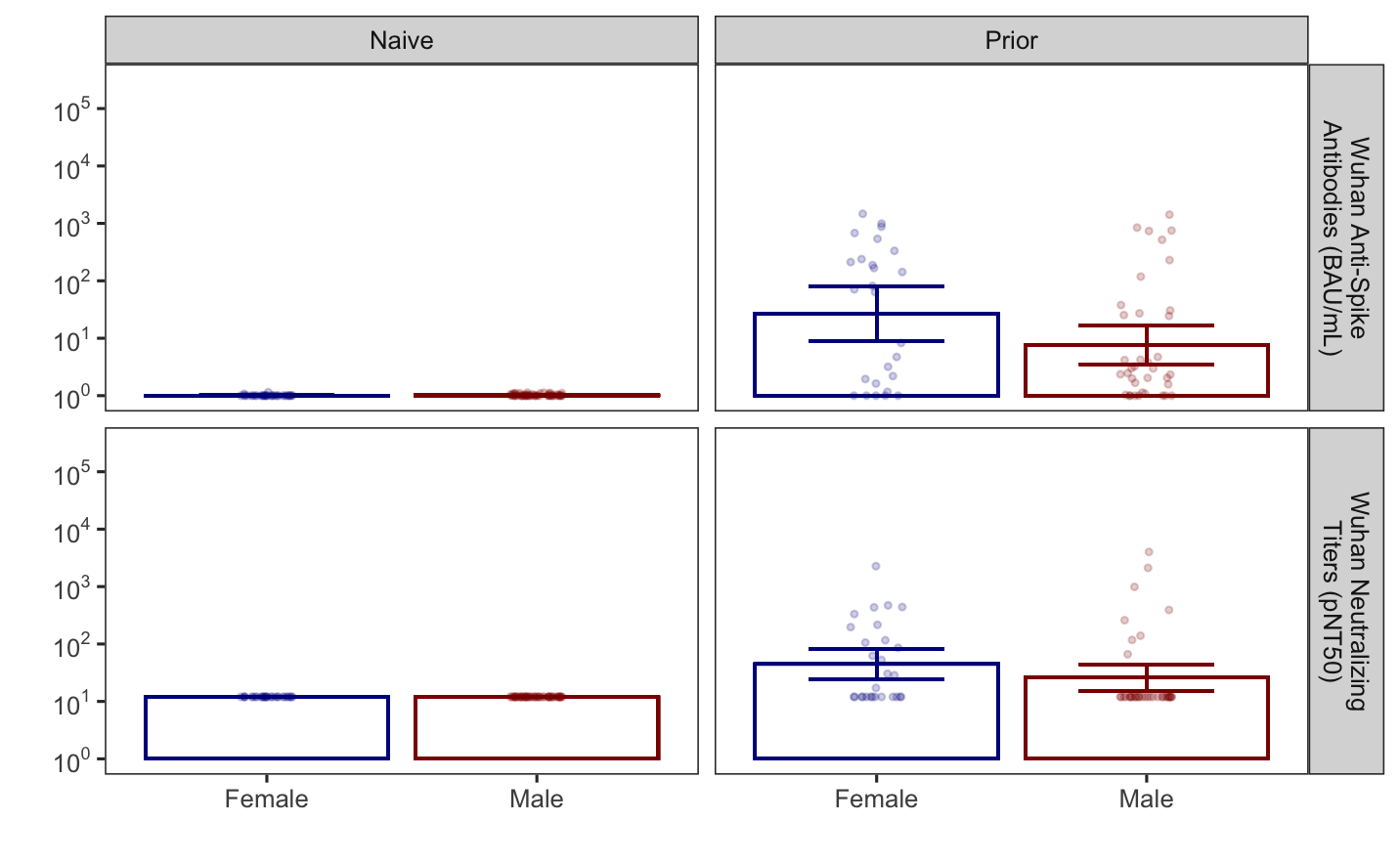


**Pre-vaccine Anti-Spike and Neutralizing Antibody titers against Wuhan strains among female and male NHR.** The bar graph shows the pre-vaccination anti-spike and neutralizing antibody titers against the Wuhan and Omicron strains among N = 151 NHR, stratified by sex and prior infection status. Wuhan anti-spike is measured in BAU/mL. The lower limit of detection (LLD) of the neutralization assay was 1:12, while the upper limit was 1:8748. Bars and whiskers show GMT with 95% CI. All infection-naive subjects were at the LLD for both assays and sex comparisons were not performed. T-tests comparing log-transformed titers between sexes were performed among subjects with prior infection and differences were not detected (Anti-Spike p-value = 0.17; Neutralizing Titer p-value = 0.063). These comparisons were hindered by the LLD.
